# Low Serum 25-Hydroxyvitamin D Levels Are Associated with Dry Eye Syndrome

**DOI:** 10.1371/journal.pone.0147847

**Published:** 2016-01-25

**Authors:** Sam Young Yoon, Seok Hyun Bae, Young Joo Shin, Shin Goo Park, Sang-Hee Hwang, Joon Young Hyon, Won Ryang Wee

**Affiliations:** 1 Department of Ophthalmology, Hallym University College of Medicine, Seoul, Republic of Korea; 2 Department of Occupational and Environmental Medicine, Inha University School of Medicine, Incheon, Republic of Korea; 3 Department of Dentistry, Keimyung University School of Medicine, Daegu, Republic of Korea; 4 Department of Ophthalmology, Seoul National University College of Medicine, Seoul, Republic of Korea; 5 Department of Ophthalmology, Seoul National University Bundang Hospital, Seongnam, Gyeonggi, Korea; University of Illinois at Chicago, UNITED STATES

## Abstract

**Background:**

Dry eye syndrome (DES) is a common tear film and ocular surface disease that results in discomfort, visual disturbance, and tear film instability with potential damage to the ocular surface. Systemic diseases associated with DES include diabetes mellitus, rheumatoid arthritis, depression, anxiety, thyroid disease, allergic diseases, irritable bowel syndrome, chronic pain syndrome, and hyperlipidemia. Interestingly, it has been found that most of these are associated with low levels of serum 25-hydroxyvitamin D (25(OH)D) or inadequate sunlight exposure.

**Methods:**

In this cross-sectional data analysis, noninstitutionalized adults aged ≥19 years (N = 17,542) who participated in Korean National Health and Nutrition Examination Survey 2010–2012 were included. Information regarding duration of sunlight exposure was collected from the survey participants. Serum 25(OH)D and zinc levels were measured. The confounding variables were age, gender, sunlight exposure time, region of residence, obesity, serum 25(OH)D level, diabetes mellitus, rheumatoid arthritis, depression, thyroid disorder, atopic dermatitis, history of ocular surgery, regular exercise, and walking exercise.

**Results:**

Mean serum 25(OH)D levels of subjects with and without DES were 16.90 ± 6.0 and 17.52 ± 6.07 (p<0.001). Inadequate sunlight exposure time (odds ratio [OR], 1.554; 95% confidence interval [CI], 1.307–1.848), urban residence (OR, 1.669; 95% CI, 1.456–1.913), indoor occupation (OR, 1.578; 95% CI, 1.389–1.814), and low serum 25(OH)D level (OR, 1.158; 95% CI, 1.026–1.308) were the risk factors for DES. After adjusting for age, sex, obesity, diabetes mellitus, rheumatoid arthritis, depression, thyroid disorder, atopic dermatitis, history of ocular surgery, regular exercise, and occupation, low serum 25(OH)D level (OR, 1.178; 95% CI, 1.010–1.372) and deficient sunlight exposure time (OR, 1.383; 95% CI, 1.094–1.749) were the risk factors for diagnosed DES.

**Conclusion:**

Low serum 25(OH)D levels and inadequate sunlight exposure are associated with DES in Korean adults. These results suggest that sufficient sunlight exposure or vitamin D supplementation may be useful in DES treatment.

## Introduction

Dry eye syndrome (DES) is a common tear film and ocular surface disease that results in discomfort, visual disturbance, and tear film instability with potential damage to the ocular surface [1]. It is accompanied by increased osmolarity of the tear film and inflammation of the ocular surface [1]. DES significantly affects the quality of life owing to symptoms of pain and irritation [2]. Inflammation is recognized to play an important role in the pathogenesis of DES [3]. Chronic inflammation stimulated by the activation of innate immune components in ocular surface cells as well as increased tear osmolarity has been involved in DES [4]. DES is a multifactorial disease associated with systemic as well as local disease [1]. Blepharitis, meibomian gland dysfunction, eyelid deformity, and conjunctivochalasis are known risk factors for DES. Systemic diseases associated with DES include diabetes mellitus, rheumatoid arthritis, depression, anxiety, thyroid disease, allergic diseases, irritable bowel syndrome, chronic pain syndrome, and hyperlipidemia [5–9]. Interestingly, it has been found that most of these are associated with low levels of serum 25-hydroxyvitamin D (25(OH)D) or inadequate sunlight exposure [10–17].

Vitamin D is produced during exposure to sunlight, and is known to regulate calcium and phosphate homeostasis [10]. However, vitamin D receptor has been discovered in most tissues and cells in human body, including corneal epithelial cells [18], salivary glands [19,20], mammary glands [10], sebaceous glands [21], and immune cells [10]. Vitamin D has been suggested to play an essential role in these organs. Low serum vitamin D levels have been reported to be associated with obesity, diabetes mellitus, inflammatory bowel disease, atopic dermatitis, depression and autoimmune diseases [11–17]. Vitamin D exerts modulating effects on the immune response of both the innate and adaptive immune systems [22]. Furthermore, the active metabolite of 25(OH)D, 1, 25-dihydroxyvitamin D, has been found to regulate cytokine production and cell proliferation [23]. Serum 25(OH)D levels have been reported to be the most accurate way to measure vitamin D status statusthe body. In the kidney, 25-hydroxy vitamin D changes into an active form of the vitamin D, 1, 25-dihydroxyvitamin D [17]. A few studies have reported the association between serum vitamin D levels and dry eye syndrome has been reported [24,25]. However, those studies has a limitation of small sample size. The association of serum vitamin D levels and sunlight exposure time on DES have not been sufficiently evaluated.

In this study, we used the Korean National Health and Nutrition Examination Survey (KNHANES) to investigate the association of vitamin D deficiency and sunlight exposure time on DES in large population.

## Methods

### Study population

The Korean National Health and Nutrition Examination Survey (KNHANES) is a series of cross-sectional surveys of nationally representative samples of the civilian Korean population aged 1 year and older that are conducted annually to assess the health and nutrition status of the South Korean population. To obtain representative samples, the KNHANES uses a stratified, multistage, cluster probability sampling design according to geographical area, age, and gender. More details regarding the sampling method are provided elsewhere [11]. The main components of the overall KNHANES survey are a health interview, health examination survey, and nutrition survey. For the health interview survey, a trained interviewer asked questions directly to individuals aged ≥19 years. This study included 17,542 adults (7,434 men and 10,108 women) aged ≥19 years who met the eligibility criteria and who completed a questionnaire regarding independent risk factors and underwent slit-lamp examinations.

### Ethics statement

The KNHANES was approved by the Korean Centers for Disease Control and Prevention Institutional Review Board, and all participants provided written informed consent. This study adhered to the tenets of the Declaration of Helsinki.

### Data collection and diagnostic criteria

For accurate data collection, participants were asked whether they have had DES symptoms (self-reported) and whether they have been previously diagnosed with DES. Subjects were asked the following question: “Until now, have you ever had dry eye symptoms before; for example, dryness of the eye or a sense of irritation?” (symptoms of DES; sxDES) Then, the subjects were asked: “To date, have you ever before been diagnosed by a physician as having a dry eye (either eye)?; emphasis was placed on the phrase ‘‘by a physician”[26]. The possible responses to the question about a previous diagnosis were “No” or “Yes” (diagnosed DES; dgDES). Data collected by the 2010–2012 KNHANES were analyzed in the present study.

### Variables

The population was divided according to residential area: rural or urban. Occupation was classified on the basis of Korean Standard Classification of Occupations as follows: group 1 (managers, professionals, and related workers); group 2 (clerks); group 3 (service and sales workers); group 4 (skilled agricultural, forestry, and fishery workers); group 5 (craft, equipment, and machine operating and assembling workers); group 6 (elementary workers); and group 7 (housewives, students, and the unemployed). In this study, groups 1, 2, 3, and 7 were merged into a single group (indoor occupation) and groups 4, 5, and 6 (outdoor occupation) also were merged, because the latter groups were reported previously to have significantly higher serum 25(OH)D levels than the former groups [11]. Obesity was defined as body mass index over 30 kg/m^2^. Diabetes mellitus was defined as fasting plasma glucose 125 mg/dL and impaired fasting glucose as fasting plasma glucose 110 mg/dL [27]. Participants were asked whether they had had rheumatoid arthritis, depression, thyroid disease, atopic dermatitis, or ocular surgery. Participants who performed moderate physical activity for more than 30 min per day on more than 5 days per week and/or strenuous physical activity for more than 20 min per day on more than 3 days per week were assigned to the regular exercise group. Regular walking was defined as walking for more than 30 min per day on more than 5 days per week, as described previously [12,14]. Data on current sunlight exposure time was obtained by selecting a single answer to the following two questions: exposure time of under 5 h, or over 5 h a day.

### Measurement of serum 25-hydroxyvitamin D and zinc levels

Blood samples were collected from the antecubital veins, refrigerated immediately, transported to the central testing facility in cold storage, and analyzed within 24 h of sampling. Serum 25(OH)D levels were measured as described previously [13] and categorized as adequate (>20 ng/mL), inadequate (range, 12 to <20 ng/mL), or deficient (<12 ng/mL), according to the guidelines set by the Food and Nutrition Board of the Institute of Medicine [28].

### Statistical analyses

Statistical analyses were performed by using the SPSS Version 18.0 (SPSS Inc., IBM Software, Portsmouth, UK), and 2-sided *P*-values of less than .05 were considered statistically significant. To produce unbiased national estimates representing the general Korean population, we used KNHANES sample weights accounting for the complex sampling design to each participant [29].

Chi-square test was used for comparison of discrete variables between groups. Student's *t*-test for independent samples was used for comparison of the differences between groups. The percentage differences were calculated as the absolute value of the change in value, divided by the average of the 2 numbers, all multiplied by 100. To estimate odds ratios (ORs) of dgDES and sxDES according to serum vitamin D levels, we conducted the logistic regression analyses by using the generalized linear model for a complex survey design. The ORs and 95% confidence intervals (CIs) were calculated in the following ways: no adjustment for potential confounders; confounder adjustment for age and gender.

## Results

### General characteristics

Characteristics of the study population are shown in Table 1. Mean age of the complete study population was 50.88 ± 16.67 years. The overall prevalence of dgDES was 10.39% (95% CI, 9.93% to 10.84%), and the prevalence of sxDES in the past 3 months was 17.79% (95% CI, 17.21% to 18.36%). Mean age among participants with dgDES (51.64 ± 16.28 years) was higher compared with non-dgDES (50.79 ± 16.71 years; *p* = 0.004, independent t-test). Pearson χ^2^ test showed significant differences between age, gender, residential region, occupation, rheumatoid arthritis, depression, thyroid disease, history of ocular surgery, regular exercise, serum 25(OH)D levels, sunlight exposure time, and dgDES. In contrast to the factors that were associated with dgDES, those that were associated with sxDES were age, gender, residential region, occupation, depression, thyroid disease, history of ocular surgery, regular exercise, walking exercise, serum zinc levels, serum 25(OH)D levels, and sunlight exposure time (Table 1).

**Table 1:** General characteristics of dry eye syndrome.

			Diagnosed DES (dgDES)				Symptoms of DES (sxDES)				
			Non-dgDES	dgDES	%difference	p-value	Non-sxDES	sxDES	%difference		p-value
Total											
N (%)			15720 (89.61%)	1822 (10.39%)			13948 (82.21%)	3018 (17.79%)			
Age (y)						<0.001*					<0.001*
<50			3453 (92.62%)	275 (7.38%)			6890 (83.28%)	1383 (16.72%)			
≥50			12267 (88.80%)	1547 (11.20%)	41.12		7058 (81.19%)	1635 (18.81%)	11.76		
Gender						<0.001*					<0.001*
Male			7023 (94.47%)	411 (5.53%)			6343 (88.50%)	824 (11.50%)			
Female			8697 (86.04%)	1411 (13.96%)	86.51		7605 (77.61%)	2194 (22.39%)	64.27		
Obesity						0.430					0.058
Non-obese			7702 (89.88%)	869 (10.12%)			6868 (82.83%)	1424 (17.17%)			
Obese			3594 (89.40%)	426 (10.60%)	4.63		3160 (81.42%)	721 (18.58%)	7.89		
Diabetes mellitus						0.713					0.927
Non-DM		10078 (89.57%)		1174 (10.43%)			8935 (82.21%)	1933 (17.79%)			
IFG		2681 (89.70%)		308 (10.30%)	1.25		2381 (82.10%)	519 (17.90%)	0.62		
DM		1486 (90.22%)		161 (9.78%)	6.43		1316 (82.56%)	278 (17.44%)	1.99		
Rheumatoid arthritis						<0.001*					0.051
Non-RA		14909 (89.75%)		1702 (10.25%)			13218 (82.26%)	2851 (17.74%)			
RA		368 (84.21%)		69 (15.79%)	42.55		330 (78.57%)	90 (21.43%)	18.84		
Depression						<0.001*					<0.001*
Non-depression		13068 (90.40%)		1387 (9.60%)			11659 (83.42%)	2317 (16.58%)			
Depression		2210 (85.20%)		384 (14.80%)	42.62		1889 (75.17%)	624 (24.83%)	39.85		
Thyroid disorder						<0.001*					<0.001*
Non-thyroid disorder		14710 (90.02%)		1630 (9.98%)			13054 (82.59%)	2752 (17.41%)			
Thyroid disorder		567 (80.08%)		141 (19.92%)	66.49		493 (72.29%)	189 (27.71%)	45.66		
History of ocular surgery						<0.001*					<0.001*
No		13435 (91.02%)		1326 (8.98%)			11999 (84.00%)	2285 (16.00%)			
Yes		2130 (81.86%)		472 (18.14%)	67.55		1831 (72.72%)	687 (27.28%)	52.12		
Atopic dermatitis						0.634					0.187
Non-AD		14590 (89.64%)		1687 (10.36%)			12944 (82.25%)	2793 (17.75%)			
AD		688 (89.12%)		84 (10.88%)	4.90		604 (80.32%)	148 (19.68%)	10.31		
Region of residence						<0.001*					<0.001*
Urban		12267 (88.80%)		1547 (11.20%)			10933 (81.67%)	2453 (18.33%)			
Rural		3453 (92.62%)		275 (7.38%)	41.12		3015 (84.22%)	565 (15.78%)	14.95		
Occupation						<0.001*					<0.001*
Indoor		11091 (88.30%)		1470 (11.70%)			9821 (80.86%)	2325 (19.14%)			
Outdoor		4128 (93.29%)		297 (6.71%)	54.21		3676 (85.83%)	607 (14.17%)	29.84		
Sunlight exposure time (h)						<0.001*					<0.001*
Sufficient (≥ 5)		2194 (93.24%)		159 (6.76%)			1906 (84.34%)	354 (15.66%)			
Inadequate (2–5)		3707 (90.68%)		381 (9.32%)	31.84		3292 (83.32%)	659 (16.68%)	6.31		
Deficient (< 2)		9790 (88.45%)		1278 (11.55%)	52.32		8722 (81.35%)	2000 (18.65%)	17.43		
Regular exercise						0.043*					0.014*
No		13929 (89.44%)		1644 (10.56%)			12336 (81.93%)	2721 (18.07%)			
Yes		1318 (91.15%)		128 (8.85%)	17.62		1182 (84.55%)	216 (15.45%)	15.63		
Walking exercise						0.062					0.048*
No		9431 (89.24%)		1137 (10.76%)			8340 (81.68%)	1871 (18.32%)			
Yes		5800 (90.15%)		634 (9.85%)	8.83		5164 (82.90%)	1065 (17.10%)	6.89		
Serum zinc level (μg/dL)						0.091					0.004*
>110		742 (45.00%)		907 (55.00%)			624 (79.29%)	163 (20.71%)			
<110		89 (51.74%)		83 (48.26%)	13.05		801 (84.67%)	145 (15.33%)	29.86		
Serum 25(OH)D level (ng/mL)						<0.001*					0.001*
Adequate (≥20)		4390 (91.29%)		419 (8.71%)			3866 (83.68%)	754 (16.32%)			
Inadequate (12–20)		7892 (89.38%)		938 (10.62%)	19.76		7071 (82.35%)	1516 (17.65%)	7.83		
Deficient (<12)		2601 (60.39%)		1706 (39.61%)	127.89		2271 (80.13%)	563 (19.87%)	19.62		

*statistically significant by chi-square test

DES = dry eye syndrome; DM = diabetes mellitus; IFG = impaired fasting glucose; RA = rheumatoid arthritis; AD = atopic dermatitis

In a binary logistic regression analysis, the risk factors for dgDES included older age (odds ratio [OR], 1.179; 95% confidence interval [CI], 1.070–1.300), female gender (OR, 2.772; 95% CI, 2.473–3.108), rheumatoid arthritis (OR, 1.642; CI, 1.264–2.134), depression (OR, 1.637; 95% CI, 1.450–1.849), thyroid disease (OR, 2.244; 95% CI, 1.853–2.718), history of ocular surgery (OR, 1.284; 95% CI, 1.166–1.414), urban residence (OR, 1.583; 95% CI, 1.385–1.810), indoor occupation (OR, 1.841; 95% CI, 1.618–2.096), low serum 25(OH)D levels (<20 ng/mL; OR, 1.280; 95% CI, 1.139–1.439), and inadequate sunlight exposure time (OR, 1.696; 95% CI, 1.433–2.008). Regular exercise (OR, 0.823; 95% CI, 0.681–0.994) was a protective factor for dgDES. The risk factors for sxDES included older age (OR, 1.154; 95% CI, 1.066–1.249), female gender (OR, 2.221; 95% CI, 2.036–2.422), depression (OR, 1.662; 95% CI, 1.503–1.839), thyroid disease (OR, 1.818; 95% CI, 1.530–2.161), history of ocular surgery (OR, 1.099; 95% CI, 1.035–1.166), urban residence (OR, 1.197; 95% CI, 1.083–1.323), indoor occupation (OR, 1.433; 95% CI, 1.300–1.580), serum 25(OH)D levels (<12 ng/mL; OR, 1.271; 95% CI, 1.126–1.434), <110 μg/dL zinc levels (OR, 1.443; 95% CI, 1.127–1.848), and inadequate sunlight exposure time (OR, 1.696; 95% CI, 1.433–2.008). Regular exercise (OR, 0.828; 95% CI, 0.712–0.963) and walking exercise (OR, 0.919; 95% CI, 0.846–0.999) were the protective factors for sxDES.

### Differences in mean serum 25-hydroxtvitamin D and zinc levels based on the presence of DES

From this adult Korean population, we determined whether serum levels of 25(OH)D or zinc correlated with dgDES or sxDES by comparing the estimated mean values. Without adjusting for potential confounders, the mean 25(OH)D levels were significantly lower in participants with dgDES or sxDES (p < 0.001 for both, independent *t*-test; Table 2). The mean serum zinc level was not different in participants with dgDES, but it was lower in participants with sxDES (p = 0.001, Table 2).

**Table 2:** Serum 25(OH)D levels and zinc levels between groups.

	Diagnosed DES (dgDES)			Symptoms of DES (sxDES)		
	Non-dgDES	dgDES	p-value	Non-sxDES	sxDES	p-value
Serum 25(OH)D level (ng/mL)	17.52 ± 6.07	16.90 ± 6.08	<0.001*	17.51 ± 6.03	17.12 ± 6.13	0.001*
Serum zinc levels (μg/dL)	136.05 ± 29.58	131.80 ± 25.79	0.070	137.09 ± 29.45	131.20 ± 28.63	0.001*

*statistically significant by independent t-test

25-hydroxyvitamin D = 25(OH)D; DES = dry eye syndrome

Serum 25(OH)D levels were lower in participants with the following characteristics: younger age, female gender, non-diabetes mellitus, rheumatoid arthritis, no history of ocular surgery, atopic dermatitis, urban residence, indoor occupation, low serum zinc levels, or inadequate sunlight exposure time (*p* < 0.001 for all except for rheumatoid arthritis, p = 0.041 for rheumatoid arthritis, independent *t*-test; Table 3) and higher in participants with regular exercise or walking exercise (*p* < 0.001 for both; Table 3).

**Table 3:** Serum 25(OH)D level (ng/mL).

	Serum 25(OH)D level (ng/mL)	p-value
Age (y)		<0.001*
<50	16.25 ± 5.26	
>50	18.70 ± 6.46	
Gender		<0.001*
Male	18.45 ± 5.97	
Female	16.49 ± 5.80	
Obesity		0.660
Non-obese	17.37 ± 5.95	
Obese	17.32 ± 6.02	
Diabetes mellitus		<0.001*
Non-DM	17.20 ± 5.94	
IFG	18.19 ± 6.09	
DM	18.25 ± 6.65	
Rheumatoid arthritis		0.041*
Non-RA	17.46 ± 6.06	
RA	18.15 ± 6.72	
Depression		0.718
Non-depression	17.49 ± 6.01	
Depression	17.44 ± 6.18	
Thyroid disorder		0.405
Non-thyroid disorder	17.47 ± 6.06	
Thyroid disorder	17.67 ± 6.31	
History of ocular surgery		<0.001*
No	17.39 ± 5.99	
Yes	17.91 ± 6.58	
Atopic dermatitis		<0.001*
Non-AD	17.52 ± 6.09	
AD	16.69 ± 5.58	
Region of residence		<0.001*
Rural	19.16 ± 6.47	
Urban	16.91 ± 5.74	
Occupation		<0.001*
Groups 4, 5 and 6 (outdoor)	19.22 ± 6.48	
Groups 1,2,3, and 7 (indoor)	16.73 ± 5.73	
Sunlight exposure time (h)		<0.001*
≥5	20.16 ± 6.55	
2–5	17.83 ± 6.12	
<2	16.77 ± 5.78	
Regular exercise		<0.001*
No	17.31 ± 5.99	
Yes	18.66 ± 6.46	
Walking exercise		<0.001*
No	17.25 ± 5.60	
Yes	17.71 ± 6.16	
Serum zinc levels (μg/dL)		<0.001*
≥110	18.61 ± 6.49	
<110	16.78 ± 5.81	

* statistically significant by independent t-test

DES = dry eye syndrome; DM = diabetes mellitus; IFG = impaired fasting glucose; RA = rheumatoid arthritis; AD = atopic dermatitis

### Factors associated with developing DES

Table 4 shows the binary logistic regression analysis between dgDES and sxDES and potential risk factors adjusted for age and gender. The analysis showed that dgDES was associated with urban residence (OR, 1.669; 95% CI, 1.456–1.913), indoor occupation (OR, 1.587; 95% CI, 1.389–1.815), depression (OR, 1.059; 95% CI, 1.008–1.113), history of ocular surgery (OR, 1.203; 95% CI, 1.103–1.312), low serum 25(OH)D level (OR, 1.158; 95% CI, 1.026–1.308), and inadequate sunlight exposure time (OR, 1.554; 1.307–1.848). Further, it showed that sxDES was associated with urban residence (OR, 1.243; 95% CI, 1.122–1.377), indoor occupation (OR, 1.264; 95% CI, 1.143–1.399), obesity (OR, 1.115; 95% CI, 1.008–1.232), depression (OR, 1.075; 95% CI, 1.032–1.120), thyroid disease (OR, 1.046; 95% CI, 1.001–1.094), history of ocular surgery (OR, 1.070; 95% CI, 1.011–1.131), and regular exercise (OR, 0.843; 95% CI, 0.724–0.982).

**Table 4:** Factors that affect the occurrence of dry eye syndrome after adjusting for age and gender.

	Diagnosed DES			Symptoms of DES		
	OR	95% CI	p-value	OR	95% CI	p-value
Region of residence						
Rural	1.0 (ref)			1.0 (ref)		
Urban	1.669	1.456–1.913	<0.001*	1.243	1.122–1.377	<0.001*
Occupation						
Outdoor	1.0 (ref)			1.0 (Ref)		
Indoor	1.587	1.389–1.815	<0.001*	1.264	1.143–1.399	<0.001*
Obesity						
Non-obese	1.0 (ref)			1.0 (ref)		
Obese	1.069	0.944–1.210	0.291	1.115	1.008–1.232	0.034*
Diabetes mellitus						
Non-DM	1.0 (ref)			1.0 (ref)		
IFG	1.067	0.929–1.224	0.359	1.075	0.962–1.202	0.200
DM	0.955	0.796–1.145	0.618	0.998	0.864–1.155	0.984
Rheumatoid arthritis						
Non-RA	1.0 (ref)			1.0 (ref)		
RA	1.026	0.969–1.085	0.380	1.025	0.978–1.074	0.299
Depression						
Non-depression	1.0 (ref)			1.0 (ref)		
Depression	1.059	1.008–1.113	0.024*	1.075	1.032–1.120	0.001*
Thyroid disorder						
Non-thyroid disorder	1.0 (ref)			1.0 (ref)		
Thyroid disorder	1.053	1.000–1.109	0.051	1.046	1.001–1.094	0.045*
History of ocular surgery						
No	1.0 (ref)			1.0 (ref)		
Yes	1.203	1.103–1.312	<0.001*	1.070	1.011–1.131	0.019*
Atopic dermatitis						
Non-AD	1.0 (ref)			1.0 (ref)		
AD	1.124	0.888–1.423	0.330	1.199	0.994–1.446	0.058
Sun exposure time (h)						
Sufficient (≥2)	1.0 (ref)			1.0 (ref)		
Inadequate (2–5)	1.389	1.143–1.690	0.001*	1.047	0.907–1.209	0.528
Deficient (<2)	1.630	1.367–1.944	<0.001*	1.119	0.984–1.271	0.085
Regular exercise						
No	1.0 (ref)			1.0 (ref)		
Yes	0.841	0.696–1.108	0.076	0.843	0.724–0.982	0.029*
Walking exercise						
No	1.0 (ref)			1.0 (ref)		
Yes	0.959	0.865–1.064	0.434	0.961	0.884–1.045	0.353
Serum 25(OH)D level (ng/mL)						
Adequate (≥20)	1.0 (ref)			1.0 (ref)		
Inadequate (12–20)	1.167	1.030–1.321	0.015*	1.036	0.939–1.143	0.483
Deficient (<12)	1.205	1.031–1.407	0.019*	1.113	0.982–1.262	0.094
Serum zinc level (μg/dL)						
≥110	1.0 (ref)			1.0 (ref)		
<110	0.926	0.671–1.278	0.638	0.798	0.620–1.029	0.081

*statistically significant by logistic regression analysis

DES = dry eye syndrome; OR = odds ratio; CI = Confidence interval; DM = diabetes mellitus; IFG = impaired fasting glucose; RA = rheumatoid arthritis; AD = atopic dermatitis

After adjusting for age, sex, obesity, diabetes mellitus, rheumatoid arthritis, depression, thyroid disorder, atopic dermatitis, history of ocular surgery, regular exercise, and occupation, low serum 25(OH)D levels were the risk factors for dgDES, but not for sxDES (OR, 1.105; 95% CI, 1.007–1.213). Inadequate (OR, 1.178; 95% CI, 1.010–1.372) and deficient serum 25(OH)D level (OR, 1.216; 95% CI, 1.007–1.469) were the risk factors for dgDES (Table 5).

**Table 5:** Relationship of serum 25-hydroxyvitamin D level and dry eye syndrome.

	Diagnosed DES			Symptoms of DES		
	OR	95% CI	p-value	OR	95% CI	p-value
Sunlight exposure time (h)						
Sufficient (≥2)	1.0 (ref)			1.0 (ref)		
Inadequate (2–5)	1.142	0.882–1.479	0.314	0.978	0.809–1.182	0.815
Deficient (<2)	1.383	1.094–1.749	0.007*	1.050	0.885–1.246	0.575
Serum 25(OH)D level (ng/mL)						
Adequate (≥20)	1.0 (ref)			1.0 (ref)		
Inadequate (12–20)	1.178	1.010–1.372	0.036*	1.045	0.926–1.179	0.475
Deficient (<12)	1.216	1.007–1.469	0.043*	1.115	0.957–1.298	0.163

Adjusted by age, sex, obesity, diabetes mellitus, rheumatoid arthritis, depression, thyroid disorder, atopic dermatitis, history of ocular surgery, regular exercise, occupation and residence. 25-hydroxyvitamin D = 25(OH)D; DES = dry eye syndrome; OR = odds ratio; CI = Confidence interval

*statistically significant by logistic regression analysis

## Discussion

DES is a common ocular disease in the general population and affects vision-related quality of life [1,30]. DES has been associated with various factors [7,30]. However, the associations between serum 25(OH)D levels and DES or between sunlight exposure time and DES have not been investigated.

In this study, we found that older age, female gender, rheumatoid arthritis, depression, thyroid disorder, history of ocular surgery, urban residence, indoor occupation, inadequate sunlight exposure, and low serum 25(OH)D level were the risk factor for dgDES. Older age and female gender are relatively well-known risk factors [6,7]. Aging, which is an important risk factor for DES, has been reported to be associated with lacrimal dysfunction [31]. Low estrogen levels after menopause in women have been described as being a major contributing factor for DES [32]. Androgen, estrogen, and progesterone receptor mRNAs have been identified in the eye [32]. Sex hormones are known to influence the immune system, and estrogen itself may modulate a cascade of inflammatory events that underlie DES [33]. Rheumatoid arthritis, depression, thyroid disorder, and history of ocular surgery are the well-known risk factors for DES [5–9,26]. DES is a common ocular manifestation in rheumatoid arthritis patients even though the severity of DES is independent of rheumatoid arthritis activity [34]. The association of DES and Graves’ ophthalmopathy have been described to suggest the mechanical impairment of orbital muscles and immune-mediated lacrimal gland dysfunction [35]. Depression is not only a risk factor for DES but anticholinergic effects of anti-depressant also can induce DES [36]. Ocular surgery induces the changes in corneal innervation which play an essential role in the pathogenesis of dry eye syndrome [37].

Urban residence and indoor occupation were the risk factors for dgDES and sxDES. In previous studies, urban dwellers and subjects with indoor occupations were reported to be factors associated with DES [8,38,39]. They have been described to be due to low indoor humidity [40], air pollution [9,41], and visual display terminal (VDT) syndrome, which is accompanied by lower blinking rates and increased tear evaporation [42]. However, the reason for the prevalence of DES in urban residency and among those with indoor occupations has not been understood thoroughly. We noticed that the participants with both urban residence and indoor occupations were exposed to sunlight inadequately. Thus, we evaluated the associations between sunlight exposure time and dgDES or sxDES. We found that inadequate sunlight exposure time (<5 h) was a risk factor for dgDES, but not for sxDES. It has been reported that sunlight exposure should be avoided, because it contributes to an increased risk of dry age-related macular degeneration, cataract, and pterygium [43]. However, sunlight exposure has been reported to prevent the development of myopia [44], depression [45,46], anxiety [16,47], diabetes [15], and autoimmune disease [48]. Sunlight exposure is by far the most important source of vitamin D [15,49]. The human skin has a large capacity for vitamin D production [49]. Vitamin D_3_ is synthesized in the skin from 7-dehydrocholesterol under the influence of UV-B (wavelength, 290–315 nm) radiation and temperature [50]. In this study, serum 25(OH)D levels were lower in DES compared to non-DES. After adjusting for age and gender, inadequate serum 25(OH)D levels (<20 ng/mL) were a risk factor with dgDES. However, sxDES was associated only with deficient serum 25(OH)D levels (<12 ng/mL), not with inadequate levels (<20 ng/mL). Vitamin D receptor has been reported to exist in ocular barrier cells [18]. It has been suggested that vitamin D might have a role in immune regulation and barrier function in ocular barrier epithelial cells [18]. Vitamin D has been reported to enhance corneal epithelial barrier function [51] through regulating gap junctions [52] and tight junction [53]. Vitamin D exerts an immunomodulating effect on the immune system [14]. In mice, vitamin D has been shown to suppress ocular surface inflammation by inhibiting Langerhans cell migration into corneas [54], thus inhibiting corneal neovascularization [55]. Furthermore, salivary glands and their epithelial and myoepithelial cells are major vitamin D targets [19], and fluid and electrolyte secretion from the parotid gland is dependent on vitamin D directly [20]. Thus, lacrimal glands as well as corneal epithelial cells also may be affected by vitamin D. It has been reported that vitamin D deficiency is prevalent in patients with Sjogren's syndrome and that female patients with Sjogren's syndrome are at risk for vitamin D deficiency [56].

In this study, we evaluated the effect of exercise on DES. After adjusting for age and gender, regular and walking exercise did not affect dgDES. However, regular exercise was a protective factor for sxDES. Regular exercise has an acute effect on salivary hormone response [57]. Regular exercise may be helpful in reducing symptoms in patients with DES. Serum zinc levels, in this study, were lower in sxDES group compared to non-sxDES group. Zinc has a role in retinal metabolism and may be beneficial in macular degeneration [58]. Zinc ion-dependent B-cell epitope is associated with primary Sjogren's syndrome [59]. However, after adjusting for age and gender, serum zinc levels did not affect sxDES. The discrepancy between the sunlight exposure and serum 25(OH)D level has been reported [60]. Serum vitamin D status can be remained despite abundant sun exposure [60]. Vitamin D obtained from the diet or cutaneous synthesis is readily taken up by adipose tissue [61]. Bioavailability of vitamin D has been reported to be reduced in obesity [62]. Thus, obesity should be considered as an confounding factor for evaluation of the effect of vitamin D. After adjusting age, gender, diabetes mellitus, rheumatoid arthritis, depression, thyroid disorder, atopic dermatitis, history of ocular surgery, regular exercise, obesity, and occupation, deficient sunlight exposure and inadequate and deficient serum 25(OH)D levels were a risk factor for DES.

In conclusion, deficient sunlight exposure time and inadequate serum 25(OH)D levels are associated with DES in Korean adults. These results suggest that sufficient sunlight exposure and/or vitamin D supplementation may be helpful in treatment of DES.

## Supporting Information

S1 Dataset Dataset containing the KNHANES (2010–2012). (XLS)

## References

[1] The definition and classification of dry eye disease: report of the definition and classification subcommittee of the international dry eye workshop (2007). Ocul Surf 2007;5:75–92. 17508116 10.1016/s1542-0124(12)70081-2

[2] Buchholz P, Steeds CS, Stern LS, Wiederkehr DP, Doyle JJ, Katz LM, et al Utility assessment to measure the impact of dry eye disease. Ocul Surf 2006; 4:155–61. 16900272 10.1016/s1542-0124(12)70043-5

[3] Stevenson W, Chauhan SK, Dana R. Dry eye disease: an immune mediated ocular surface disorder. Arch Ophthalmol 2012; 130:90–100. 10.1001/archophthalmol.2011.364 22232476 PMC3677724

[4] Barabino S, Chen Y, Chauhan S, Dana R. Ocular surface immunity: homeostastic mechanisms and their disruption in dry eye disease. Prog Retin Eye Res 2012; 31:271–285. 10.1016/j.preteyeres.2012.02.003 22426080 PMC3334398

[5] Vehof J, Kozareva D, Hysi PG, Hammond CJ. Prevalence and risk factors of dry eye disease in a British female cohort. Br J Ophthalmol 2014;98:1712–7. 10.1136/bjophthalmol-2014-305201 25185440

[6] Lee AJ, Lee J, Saw SM, Gazzard G, Koh D, Widjaja D, et al Prevalence and risk factors associated with dry eye symptoms: a population based study in Indonesia. Br J Ophthalmol 2002;86:1347–51. 12446361 10.1136/bjo.86.12.1347 PMC1771386

[7] Schaumberg DA, Sullivan DA, Dana MR. Epidemiology of dry eye syndrome. Adv Exp Med Biol. 2002;506:989–998. 12614022 10.1007/978-1-4615-0717-8_140

[8] Han SB, Hyon JY, Woo SJ, Lee JJ, Kim TH, Kim KW. Prevalence of dry eye disease in an elderly Korean population. Arch Ophthalmol 2011;129:633–8. 10.1001/archophthalmol.2011.78 21555618

[9] Galor A, Kumar N, Feuer W, Lee DJ. Environmental factors affect the risk of dry eye syndrome in a United States veteran population. Ophthalmology 2014;121:972–3. 10.1016/j.ophtha.2013.11.036 24560568

[10] Holick MF. Vitamin D deficiency. N Engl J Med 2007;357:266–81. 17634462 10.1056/NEJMra070553

[11] Rhee SY, Hwang YC, Chung HY, Woo JT. Vitamin D and diabetes in Koreans: analyses based on the Fourth Korea National Health and Nutrition Examination Survey (KNHANES), 2008–2009. Diabet Med 2012;29:1003–10. 10.1111/j.1464-5491.2012.03575.x 22247968 PMC3504347

[12] Choi HS, Oh HJ, Choi H, Choi WH, Kim JG, Kim KM, et al Vitamin D insufficiency in Korea–a greater threat to younger generation: the Korea National Health and Nutrition Examination Survey (KNHANES) 2008. J Clin Endocrinol Metab 2011;96:643–51. 10.1210/jc.2010-2133 21190984

[13] Nam GE, Kim DH, Cho KH, Park YG, Han KD, Kim SM, et al 25-Hydroxyvitamin D insufficiency is associated with cardiometabolic risk in Korean adolescents: the 2008–2009 Korea National Health and Nutrition Examination Survey (KNHANES). Public Health Nutr 2014;17:186–94. 10.1017/S1368980012004855 23168294 PMC10282263

[14] Cheng HM, Kim S, Park GH, Chang SE, Bang S, Won CH, et al Low vitamin D levels are associated with atopic dermatitis, but not allergic rhinitis, asthma, or IgE sensitization, in the adult Korean population. J Allergy Clin Immunol 2014;133:1048–55. 10.1016/j.jaci.2013.10.055 24388009

[15] Geldenhuys S, Hart PH, Endersby R, Jacoby P, Feelisch M, Weller RB, et al Ultraviolet radiation suppresses obesity and symptoms of metabolic syndrome independently of vitamin D in mice fed a high-fat diet. Diabetes 2014;63:3759–69. 10.2337/db13-1675 25342734

[16] Mosher CE, Danoff-Burg S. Addiction to indoor tanning: relation to anxiety, depression, and substance use. Arch Dermatol 2010;146:412–7. 10.1001/archdermatol.2009.385 20404230 PMC3756887

[17] Berridge MJ. Vitamin D cell signalling in health and disease. Biochem Biophys Res Commun 2015;460:53–71. 10.1016/j.bbrc.2015.01.008 25998734

[18] Alsalem JA, Patel D, Susarla R, Coca-Prados M, Bland R, Walker EA, et al Characterization of vitamin D production by human ocular barrier cells. Invest Ophthalmol Vis Sci 2014;55:2140–7. 10.1167/iovs.13-13019 24576880

[19] Stumpf WE, Hayakawa N. Salivary glands epithelial and myoepithelial cells are major vitamin D targets. Eur J Drug Metab Pharmacokinet 2007;32:123–9. 18062404 10.1007/BF03190474

[20] Peterfy C, Tenenhouse A, Yu E. Vitamin D and parotid gland function in the rat. J Physiol 1988;398:1–13. 3392666 10.1113/jphysiol.1988.sp017025 PMC1191755

[21] Kramer C, Seltmann H, Seifert M, Tilgen W, Zouboulis CC, Reichrath J. Characterization of the vitamin D endocrine system in human sebocytes in vitro. J Steroid Biochem Mol Biol 2009;113:9–16. 10.1016/j.jsbmb.2008.10.010 19027855

[22] Muehleisen B, Gallo RL. Vitamin D in allergic disease: shedding light on a complex problem. J Allergy Clin Immunol 2013;131:324–9. 10.1016/j.jaci.2012.12.1562 23374263

[23] Consiglio M, Viano M, Casarin S, Castagnoli C, Pescarmona G, Silvagno F. Mitochondrial and lipogenic effects of vitamin D in differentiating and proliferating human keratinocytes. Exp Dermatol 2015 5 23. 10.1111/exd.12761 26010336

[24] Galor A, Gardener H, Pouyeh B, Feuer W, Florez H. Effect of a Mediterranean dietary pattern and vitamin D levels on Dry Eye syndrome. Cornea. 2014;33:437–41. 10.1097/ICO.0000000000000089 24622300 PMC3972326

[25] Kurtul BE, Özer PA, Aydinli MS. The association of vitamin D deficiency with tear break-up time and Schirmer testing in non-Sjögrendry eye. Eye (Lond). 2015;29:1081–4. 26066054 10.1038/eye.2015.96 PMC4541362

[26] Ahn JM, Lee SH, Rim TH, Park RJ, Yang HS, Kim TI, et al Prevalence of and risk factors associated with dry eye: the Korea National Health and Nutrition Examination Survey 2010–2011. Am J Ophthalmol 2014;158:1205–1214. 10.1016/j.ajo.2014.08.021 25149910

[27] Nichols GA, Hillier TA, Brown JB. Progression from newly acquired impaired fasting glusose to type 2 diabetes. Diabetes Care. 2007;30:228–33. 17259486 10.2337/dc06-1392 PMC1851903

[28] Institute of Medicine of the National Academies. Dietary reference intakes for calcium and vitamin D Washington, DC: The National Academies Press; 2010.

[29] Choi CJ, Seo M, Choi WS, Kim KS, Youn SA, Lindsey T, et al Relationship between serum 25-hydroxyvitamin D and lung function among Korean adults in Korea National Health and Nutrition Examination Survey (KNHANES), 2008–2010. J Clin Endocrinol Metab 2013;98:1703–10. 10.1210/jc.2012-3901 23533242

[30] Miljanović B, Dana R, Sullivan DA, Schaumberg DA. Impact of dry eye syndrome on vision-related quality of life. Am J Ophthalmol 2007;143:409–15. 17317388 10.1016/j.ajo.2006.11.060 PMC1847608

[31] Kawashima M, Kawakita T, Inaba T, Okada N, Ito M, Shimmura S, et al Dietary lactoferrin alleviates age-related lacrimal gland dysfunction in mice. PLoS One 2012;7:e33148 10.1371/journal.pone.0033148 22479365 PMC3314001

[32] Wickham LA, Gao J, Toda I, Rocha EM, Ono M, Sullivan DA. Identification of androgen, estrogen and progesterone receptor mRNAs in the eye. Acta Ophthalmol Scand 2000;78:146–53. 10794246 10.1034/j.1600-0420.2000.078002146.x

[33] Truong S, Cole N, Stapleton F, Golebiowski B. Sex hormones and the dry eye. Clin Exp Optom 2014;97:324–36. 10.1111/cxo.12147 24689906

[34] Fujita M, Igarashi T, Kurai T, Sakane M, Yoshino S, Takahashi H. Correlation between dry eye and rheumatoid arthritis activity. Am J Ophthalmol 2005;140:808–13. 16289424 10.1016/j.ajo.2005.05.025

[35] Selter JH, Gire AI, Sikder S. The relationship between Graves' ophthalmopathy and dry eye syndrome. Clin Ophthalmol 2014;9:57–62. 10.2147/OPTH.S76583 25584018 PMC4287254

[36] Wong J, Lan W, Ong LM, Tong L. Non-hormonal systemic medications and dry eye. Ocul Surf 2011;9212–26. 10.1016/s1542-0124(11)70034-9 22023816

[37] Chao C, Golebiowski B, Stapleton F. The role of corneal innervation in LASIK-induced neuropathic dry eye. Ocul Surf 2014;12:32–45. 10.1016/j.jtos.2013.09.001 24439045

[38] Jie Y, Xu L, Wu YY, Jonas JB. Prevalence of dry eye among adult Chinese in the Beijing Eye Study. Eye (Lond) 2009;23:688–93. 18309341 10.1038/sj.eye.6703101

[39] Goto E, Yagi Y, Matsumoto Y, Tsubota K. Impaired functional visual acuity of dry eye patients. Am J Ophthalmol 2002;133:181–186. 11812420 10.1016/s0002-9394(01)01365-4

[40] Wolkoff P, Kjaergaard SK. The dichotomy of relative humidity on indoor air quality. Environ Int 2007;33:850–7. 17499853 10.1016/j.envint.2007.04.004

[41] Lois N, Abdelkader E, Reglitz K, Garden C, Ayres JG. Environmental tobacco smoke exposure and eye disease. Br J Ophthalmol 2008;92:1304–10. 10.1136/bjo.2008.141168 18658170

[42] Uchino M, Schaumberg DA, Dogru M, Uchino Y, Fukagawa K, Shimmura S, et al Prevalence of dry eye disease among Japanese visual display terminal users. Ophthalmology 2008;115:1982–8. 10.1016/j.ophtha.2008.06.022 18708259

[43] Dolin PJ, Johnson GJ. Solar ultraviolet radiation and ocular disease: a review of the epidemiological and experimental evidence. Ophthalmic Epidemiol 1994;1:155–64. 8790622 10.3109/09286589409047224

[44] Wang Y, Ding H, Stell WK, Liu L, Li S, Liu H, et al Exposure to sunlight reduces the risk of myopia in rhesus monkeys. PLoS One 2015;10:e0127863 10.1371/journal.pone.0127863 26030845 PMC4451516

[45] Vyssoki B, Kapusta ND, Praschak-Rieder N, Dorffner G, Willeit M. Direct effect of sunshine on suicide. JAMA Psychiatry 2014;71:1231–7. 10.1001/jamapsychiatry.2014.1198 25208208

[46] Axelsson J, Ragnarsdóttir S, Pind J, Sigbjörnsson R. Daylight availability: a poor predictor of depression in Iceland. Int J Circumpolar Health 2004;63:267–76. 10.3402/ijch.v63i3.17736 15526930

[47] Doganay Z, Sunter AT, Guz H, Ozkan A, Altintop L, Kati C, et al Climatic and diurnal variation in suicide attempts in the ED. Am J Emerg Med 2003;21:271–5. 12898481 10.1016/s0735-6757(03)00039-1

[48] Lucas RM, Ponsonby AL. Considering the potential benefits as well as adverse effects of sun exposure: can all the potential benefits be provided by oral vitamin D supplementation? Prog Biophys Mol Biol 2006;92:140–9. 16616326 10.1016/j.pbiomolbio.2006.02.019

[49] Webb AR, Pilbeam C, Hanafin N, Holick MF. An evaluation of the relative contributions of exposure to sunlight and of diet to the circulating concentrations of 25- hydroxyvitamin D in an elderly nursing home population in Boston. Am J Clin Nutr 1990; 51: 1075 81. 2349922 10.1093/ajcn/51.6.1075

[50] Lo CW, Paris PW, Holick MF. Indian and Pakistani immigrants have the same capacity as Caucasians to produce vitamin D in response to ultraviolet irradiation. Am J Clin Nutr 1986; 44: 683–85. 3766454 10.1093/ajcn/44.5.683

[51] Yin Z, Pintea V, Lin Y, Hammock BD, Watsky MA. Vitamin D enhances corneal epithelial barrier function. Invest Ophthalmol Vis Sci 2011;52:7359–64. 10.1167/iovs.11-7605 21715350 PMC3183972

[52] Lu X, Watsky MA. Effects of vitamin D receptor knockout on cornea epithelium gap junctions. Invest Ophthalmol Vis Sci 2014;55:2975–82. 10.1167/iovs.13-13788 24722695 PMC4012941

[53] Elizondo RA, Yin Z, Lu X, Watsky MA. Effect of vitamin D receptor knockout on cornea epithelium wound healing and tight junctions. Invest Ophthalmol Vis Sci 2014;55:5245–51. 10.1167/iovs.13-13553 25061117 PMC4142771

[54] Suzuki T, Sano Y, Kinoshita S. Effects of 1alpha,25-dihydroxyvitamin D3 on Langerhans cell migration and corneal neovascularization in mice. Invest Ophthalmol Vis Sci 2000;41:154–8. 10634615

[55] Suzuki T, Sano Y, Sotozono C, Kinoshita S. Regulatory effects of 1alpha,25-dihydroxyvitamin D(3) on cytokine production by human corneal epithelial cells. Curr Eye Res 2000;20:127–30. 10617914

[56] Erten Ş, Şahin A, Altunoğlu A, Gemcioğlu E, Koca C. Comparison of plasma vitamin D levels in patients with Sjögren's syndrome and healthy subjects. Int J Rheum Dis 2015; 18:70–5. 10.1111/1756-185X.12298 24467766

[57] Beaven CM, Gill ND, Ingram JR, Hopkins WG. Acute salivary hormone responses to complex exercise bouts. J Strength Cond Res 2011;25:1072–8. 10.1519/JSC.0b013e3181bf4414 20703172

[58] Brown NA, Bron AJ, Harding JJ, Dewar HM. Nutrition supplements and the eye. Eye (Lond) 1998;12 (Pt 1):127–33. 9614529 10.1038/eye.1998.21

[59] Routsias JG, Kosmopoulou A, Makri A, Panou-Pomonis E, Sakarellos C, Sakarellos-Daitsiotis M, et al Zinc ion dependent B-cell epitope, associated with primary Sjogren's syndrome, resides within the putative zinc finger domain of Ro60kD autoantigen: physical and immunologic properties. J Med Chem 2004;47:4327–34. 15294004 10.1021/jm049844+

[60] Binkley N, Novotny R, Krueger D, Kawahara T, Daida YG, Lensmeyer G, et al Low vitamin D status despite abundant sun exposure. J Clin Endocrinol Metab 2007;92:2130–5. 17426097 10.1210/jc.2006-2250

[61] Blum M, Dolnikowski G, Seyoum E, Harris SS, Booth SL, Peterson J, et al Vitamin D(3) in fat tissue. Endocrine 2008; 33: 90–94. 10.1007/s12020-008-9051-4 18338271 PMC2839878

[62] Wortsman J, Matsuoka LY, Chen TC, Lu Z, Holick MF. Decreased bioavailability of vitamin D in obesity. Am J Clin Nutr 2000;72:690–3. 10966885 10.1093/ajcn/72.3.690

